# KIAA1429 and ALKBH5 Oppositely Influence Aortic Dissection Progression *via* Regulating the Maturation of Pri-miR-143-3p in an m6A-Dependent Manner

**DOI:** 10.3389/fcell.2021.668377

**Published:** 2021-08-19

**Authors:** Peng Wang, Zhiwei Wang, Min Zhang, Qi Wu, Feng Shi, Shun Yuan

**Affiliations:** ^1^Department of Cardiovascular Surgery, Renmin Hospital of Wuhan University, Wuhan, China; ^2^Cardiovascular Surgery Laboratory, Renmin Hospital of Wuhan University, Wuhan, China; ^3^Central Laboratory, Renmin Hospital of Wuhan University, Wuhan, China

**Keywords:** aortic dissection, m6A, KIAA1429, ALKBH5, miR-143-3p

## Abstract

Despite decades of study into aortic dissection (AD), a lethal cardiovascular emergency due to a tear in the aorta intima or bleeding within the aortic wall, leading to the separation of the different layers of it, the factors that influence its progression and the deeper regulatory mechanisms remain poorly understood. Nowadays, with the maturity of N6-methyladenosine (m6A) sequence technology, m6A modification, one type of RNA epigenesis, has gradually become a new research hotspot for epigenetic molecular regulation. Especially recently, increasing evidence has revealed that m6A modification functions as a pivotal post-transcriptional modification to influence the progression of multiple diseases. Based on these findings, it is reasonable to speculate that m6A modification may affect the onset and progression of AD. To explore the validity of our conjecture and to elucidate its underlying molecular mechanism of action, we conducted the present study. In this study, we found that KIAA1429 is downregulated while ALKBH5 is upregulated in aortic tissues from AD patients. Furthermore, gain- and loss-of-function studies showed that KIAA1429 and ALKBH5 can oppositely regulate HASMC proliferation, HAEC apoptosis, and AD progression in AngII-infused mice. Mechanistically, we demonstrated that KIAA1429/ALKBH5-mediated m6A modifications can regulate the processing of pri-miR-143-3p through interacting with the microprocessor protein DGCR8, thus indirectly regulating the downstream target gene of mature miR-143-3p, DDX6, to perform their biological functions *in vitro* and *in vivo*. Our findings have revealed a novel connection between m6A modification and AD progression and may provide a novel molecular basis for subsequent researchers to search for novel therapeutic approaches to improve the health of patients struggling with AD.

## Introduction

Aortic dissection (AD) is a lethal cardiovascular emergency due to a tear in the aorta intima or bleeding within the aortic wall, leading to the separation of the different layers of the aortic wall (Gawinecka et al., 2017; Tchana-Sato et al., 2018). Despite decades of study into AD, the factors that influence its progression and the deeper regulatory mechanisms remain poorly understood. Therefore, one of the prerequisites to the development of novel coping strategies for AD is to elucidate the underlying molecular mechanisms involved.

According to the results of available studies, both decreased cell proliferation capacity of human aortic smooth muscle cells (HASMCs) and excessive apoptosis of human aortic endothelial cells (HAECs) in the arterial vascular wall are closely related to the development of AD (Lai et al., 2019; Song et al., 2019; Sun et al., 2020). Although numerous studies have confirmed this conclusion, the molecular regulatory mechanisms involved in HASMC and HAEC dysfunction are not well investigated, especially epigenetic studies. Epigenetic abnormalities mainly occur at the following levels: DNA (Cantara et al., 2011), RNA (Berger, 2002), and histone modification (Akhavan-Niaki and Samadani, 2013). At the RNA level, diverse types of posttranscriptional modifications have been recognized, among which N6-methyladenosine (m6A) RNA methylation is one of the most common modifications in eukaryote messenger RNAs (Desrosiers et al., 1974; Dominissini et al., 2012; Meyer et al., 2012).

Nowadays, with the maturity of m6A sequencing technology, m6A modification, one type of RNA epigenesis, has gradually become a new research hotspot for epigenetic molecular regulation (Frye et al., 2018; He et al., 2019). M6A modification has been confirmed to be a dynamic and reversible process modulated by m6A WERs (writers, readers, and erasers). The methyltransferase complex that catalyzes the formation of m6A methylation is called “writers,” among which methyltransferase like 3 (METTL3), methyltransferase like 14 (METTL14), Vir-like methyltransferase-associated (KIAA1429), and WT1-associated protein (WTAP) play vital roles (Lan et al., 2019). In turn, demethylase, fat mass and obesity-associated protein (FTO), and alkylation repair homolog protein 5 (ALKBH5) function as “erasers” to remove the m6A methylation (Meyer and Jaffrey, 2017). Meanwhile, several m6A-binding proteins, including YTHDC1/2, YTHDF1/2/3, and HNRNP, act as the “readers” to decode m6A methylation (Roundtree et al., 2017).

Recently, accumulating evidence has revealed that m6A modification influences the progression of multiple diseases through regulating non-coding RNA biogenesis. For instance, Zhang et al. (2019b) found that ALKBH5 could promote invasion and metastasis of gastric cancer *via* decreasing methylation of the lncRNA NEAT1. Zuo et al. (2020) reported that M6A-mediated upregulation of LINC00958 exacerbates hepatocellular carcinoma progression. Zhang et al. (2019a) demonstrated that excessive miR-25-3p expression induced by m6A promotes pancreatic cancer progression. However, the above findings mainly focus on the field of oncology, and whether m6A modification is also involved in human cardiovascular diseases by regulating non-coding RNAs biogenesis has been less reported (Su et al., 2020).

This study aims to explore the role of m6A modification in the development of AD and elucidate the underlying mechanisms by which m6A modification influences AD progression.

## Materials and Methods

### Clinical Samples

The collection and usage of clinical specimens in this study were approved by the Clinical Research Ethics Committees of Renmin Hospital of Wuhan University, China. Meanwhile, informed written consent of all participants was obtained. Aortic tissues (*n* = 25) were collected from acute thoracic AD patients who underwent emergency aortic replacement surgery from April 2018 to December 2019. Normal aorta tissues (*n* = 25) were obtained from heart donors declared brain dead. The clinical data of all donors and AD patients are present in Supplementary Table 1.

### Cell Culture and Cell Transfection

HAECs were purchased from ATCC (#PCS-100-011^TM^, Manassas, VA, United States) and cultured in Endothelial Cell Growth Basal Medium-2 (Lonza, Basel, CHE). HASMCs were purchased from ATCC (#PCS-100-012^TM^) and cultured in DMEM (HyClone, Logan, UT, United States). All media were supplemented with endothelial cell growth factors, 5% fetal bovine serum (Invitrogen, Carlsbad, CA, United States), and 1% penicillin/streptomycin (Sigma, Darmstadt, DEU). The external conditions for cell culture were as follows: 37°C, humidified atmosphere containing 5% CO_2_.

To obtain stable transfection cell lines, we transfected cells with diverse lentivirus vector (Genechem, Shanghai, China) carrying overexpression plasmids (termed as LV-KIAA1429/LV-ALKBH5), a negative control (termed as LV-NC), interfering RNA (termed as sh-KIAA1429/sh-ALKBH5), or scramble control (termed as sh-NC). Stable cell clones were selected by using puromycin (4 μg/ml) for 1 week. To interfere with miR-143-3p and DDX6 expression, miR-143-3p mimics, inhibitor, pcDNA3.1-DDX6, si-DDX6, or their respective negative controls (NC) were synthesized by GeneChem Co., Ltd. (Shanghai, China) and transiently transfected into cells using Lipofectamine 3000 reagent (Invitrogen).

### AngII Infusion AD Model and Adenovirus Vector Transfection

The Ethical Committee of the Renmin Hospital of Wuhan University approved the animal experiments, which were designed in accordance with the Wuhan Directive for Animal Research and the Current Guidelines for the Care and Use of Laboratory Animals published by the National Institutes of Health (NIH). Male C57BL/6N mice were purchased from the Guangdong Medical Laboratory Animal Center (Foshan, China). The angiotensin II (AngII)-infusion mouse AD model was established as described in our previous study (Wang et al., 2021). Concretely, osmotic mini-pumps (Alzet, Cupertino, CA, United States) containing AngII (1 μg/kg/min, Enzo Biochem, New York, NY, United States) were implanted in 7-week-old male mice. Moreover, to interfere with the expression of KIAA1429, ALKBH5, or DDX6 *in vivo*, adeno-associated virus 9 (AAV9) vectors carrying a variety of overexpression plasmids or interfering RNA were randomly injected through the tail vein to C57BL/6N mice. All mice were monitored daily to record their survival time and death reasons. The experimental endpoint was mouse death from aortic rupture or treatment time up to 28 days. The aortas of mice were collected to confirm the formation of AD.

### RNA Extraction and qRT-PCR

Total RNA was extracted from tissues and cells using Trizol Reagent (Invitrogen). Subsequent concentration determination of samples, reverse transcription, and qRT-PCR were performed as reported in our previous work (Wang et al., 2017). The expression of pri-miR-143-3p and pri-miR-3129 was examined using a TaqMan pri-miRNA assay. The primer sequences used in the present study are listed in Supplementary Table 2.

### Western Blot

Cells or tissue samples were lysed by RIPA lysis buffer containing 1% protease inhibitors (Sigma). The protein extractions were quantified by BCA Protein Assay Kit (Beyotime, Shanghai, China). Western blot assays were performed as described in our previous work (Wang et al., 2017). Primary antibodies used in this study are shown in Supplementary Table 3.

### Cell Proliferation Assays

The cell proliferation was detected by Cell Counting Kit-8 (CCK8, Dojindo, Kumamoto, Japan) and 5-ethynyl-2′-deoxyuridine (EdU; Ribobio, Guangzhou, China) assays. CCK8 assay was conducted as described in our previous work (Wang et al., 2017).

5-ethynyl-2′-deoxyuridine assay was performed in accordance with the protocol for the Click-iT^®^ EdU Imaging Kit (Invitrogen). Cells were hatched with EdU for 2 h, then fixed in 4% paraformaldehyde for 1 h, and incubated with glycine. Subsequently, the cells were washed with TBS after being submerged into the cocktail for 0.5 h in darkness and further covered in Hoechst for 30 min. Images were captured by a fluorescence microscope (BX63, Olympus, Japan).

### Cell Apoptosis Assays

In the TUNEL assay, cells were fixed in paraformaldehyde for 15 min and then stained by *in situ* Cell Death Detection Kit (TMR red) (Roche, Basel, CHE). We randomly chose six visual fields from each group to count the positive TUNEL-stained cells. Apoptotic cells were presented as percentages (cell count with positive TUNEL staining/cell count with positive DAPI staining) and we obtained images using a fluorescence microscope (BX63, Olympus).

In flow cytometry analysis, cells were collected, resuspended, and double-stained with Annexin V-FITC (#556547, BD Biosciences, Franklin, NJ, United States) as well as propidium iodide (PI; Solarbio, Beijing, China) at room temperature for 10 min in darkness. Subsequently, cell apoptosis was analyzed using the FACScan flow cytometer installed with Cell Quest software (BD Biosciences).

### RNA m6A Dot Blots

The Poly(A)^+^ RNAs (100 and 250 ng, respectively) were denatured and spotted onto a nylon membrane (Sigma) with a Bio-Dot apparatus (Bio-Rad, Hercules, CA, United States). Then, membranes were ultraviolet (UV) crosslinked, blocked, incubated with m6A antibody (#202003, Synaptic Systems, Canoga Park, CA, United States) overnight at 4°C, and hatched with HRP-conjugated anti-mouse IgG (Proteintech, Rosemont, IL, United States). After extensive washing, membranes were visualized by chemiluminescence system (Bio-Rad). Meanwhile, the membrane stained with 0.02% methylene blue (MB) in 0.3 M sodium acetate (pH 5.2) was used to show the consistency between groups.

### Dual-Luciferase Reporter Assay

The 3′-UTR of DDX6 was amplified and then inserted into the *Xba*I restriction enzyme site of a pMiR-REPORT^TM^ Luciferase vector (Promega, Madison, WI, United States). We mutated the binding site of miR-143-3p in the 3′-UTR-loading vectors using the QuikChange site-directed mutagenesis kit (Promega). The activity of firefly luciferase was detected by Dual-Luciferase Reporter Assay (Promega), being normalized to Renilla luciferase activity (Wang et al., 2017).

### Ectopic Reporter Constructs

We recomposed a previously described ectopic reporter construct (Auyeung et al., 2013; Alarcón et al., 2015) to analyze the pri-miR-143-3p processing. Concretely, we displaced pri-miR-1-1, the miRNA control, with its modified version in which the adenosine [A] of the potential RRACH m6A sequence motif was mutated. We next placed it downstream of the query miRNA, either wild-type (WT) version (WT) or mutant version (Mut) pri-miR-143-3p in which the adenosine [A] of the RRACH m6A sequence motif was mutated to thymidine [T]. Then, these two constructs were transfected into cells utilizing Lipofectamine 3000 (Invitrogen), and qRT-PCR was used to determine the production of mature miR-1-1 and miR-143-3p.

### RNA Immunoprecipitation Assay

RNA immunoprecipitation (RIP) assay was conducted using the Magna RIP^TM^ RNA-Binding Protein Immunoprecipitation Kit (Millipore, MA, United States). After cells were lysed and centrifuged, the supernatant was incubated with magnetic beads conjugated with antibodies against Argonaute-2 (anti-Ago2) or anti-Immunoglobulin G (anti-IgG) at 4°C overnight. The precipitated RNAs were eluted, purified, and then detected by qRT-PCR.

### m6A RNA Immunoprecipitation Assay

The RNAs isolated from cells were treated with DNase I (Thermo Fisher Scientific, United States) and then chemically fragmented to 100 nt on the ice. The anti-m6A antibody (1:1,000, Abcam, United States) previously combined with magnetic Dynabeads (Thermo Fisher Scientific) was used for immunoprecipitations in the RIP Immunoprecipitation buffer (Magna RIP Kit, Millipore) and incubated with DNA-free fragmented RNAs. Then, the beads were treated with Proteinase K (25 mg/ml) at 42°C for 2 h. RNAs were extracted and subjected to qRT-PCR.

### Co-immunoprecipitation Assay

Cell lysates were centrifuged and the supernatant was preincubated with 20 μg of protein A-Sepharose beads (Thermo Fisher Scientific) with vibrating for 2 h at 4°C. The samples were then centrifuged again and transferred to a new 2-ml EP tube. The samples were incubated with the primary antibodies for 1.5 h and then incubated with protein A-Sepharose beads to capture the immunocomplex. Next, the beads were washed three times, dissolved in electrophoresis sample loading buffer, and incubated for 15 min at 95°C.

### Bioinformatics Database

We screened out candidate miRNAs whose expression might be regulated by KIAA1429 or ALKBH5 using the LinkedOmics database. We searched m6AVar database for the predicted m6A sites in pri-miR-143-3p. TargetScan (context score < −0.2), miRanda (score < −0.5), and PicTar (total context + + score < −0.6) databases were used to predict the target genes of miRNA. The available addresses for the above databases are presented in Supplementary Table 4.

### Statistical Analysis

All statistical analyses were performed by using GraphPad Prism 8.0 (GraphPad, United States) and SPSS 24.0 (IBM, United States), and two-tailed Student’s *t*-test and Pearson’s correlation coefficient analysis were, respectively, adopted to analyze differences between groups or the correlations between KIAA1429, ALKBH5, miR-143-3p, and DDX6. Kaplan–Meier curve with log-rank test was utilized to analyze the survival time. A two-sided *p*-value < 0.05 was considered to be statistically significant. Each experiment was performed in triplicate, and all data are expressed as mean ± standard error of the mean (SEM).

## Results

### KIAA1429 Is Downregulated While ALKBH5 is Upregulated in Aorta Samples From AD Patients

**FIGURE 1:**
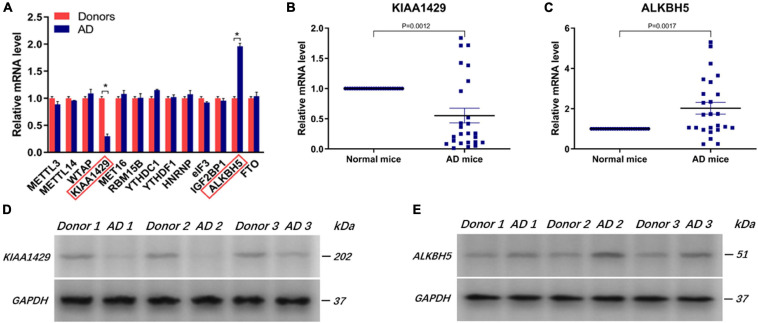
KIAA1429 is downregulated while ALKBH5 is upregulated in aorta samples from AD patients. **(A)** KIAA1429 was significantly downregulated while ALKBH5 was upregulated in the aorta specimens of AD patients compared to donors. **(B,C)** The low expression of KIAA1429 or high expression of ALKBH5 was confirmed in 25 pairs of aortic tissues from normal and AngII-induced AD mice. **(D,E)** The protein levels of KIAA1429 and ALKBH5 in aorta specimens from AD patients were significantly lower and higher than in aorta tissues from donors, respectively. Data are presented as the mean ± SEM (*N* ≥ 3 per group); *p < 0.05.

To identify whether m6A modification was involved in AD development, we initially measured the expression levels of multiple key genes related to m6A, as described in section “Introduction,” in 25 pairs of aorta tissues derived from donors and AD patients. It was observed that KIAA1429 was significantly downregulated while ALKBH5 was upregulated in the aorta specimens of AD patients compared to donors (Figure 1A and Supplementary Figure 1). Subsequently, when the expression levels of KIAA1429 and ALKBH5 were further detected in 25 pairs of aorta samples from normal and AngII-induced AD mice, the same change trends emerged (Figures 1B,C). Furthermore, the protein levels of KIAA1429 and ALKBH5 in aorta specimens from AD patients were significantly lower and higher than in aorta tissues from donors, respectively (Figures 1D,E). Taken together, the aberrant expression of KIAA1429 and ALKBH5 in aortas of AD patients indicated that m6A modification mediated by methyltransferase or demethylase might be indeed involved in AD progression.

### KIAA1429 Enhances While ALKBH5 Reduces m6A Levels in HASMCs and HAECs

**FIGURE 2:**
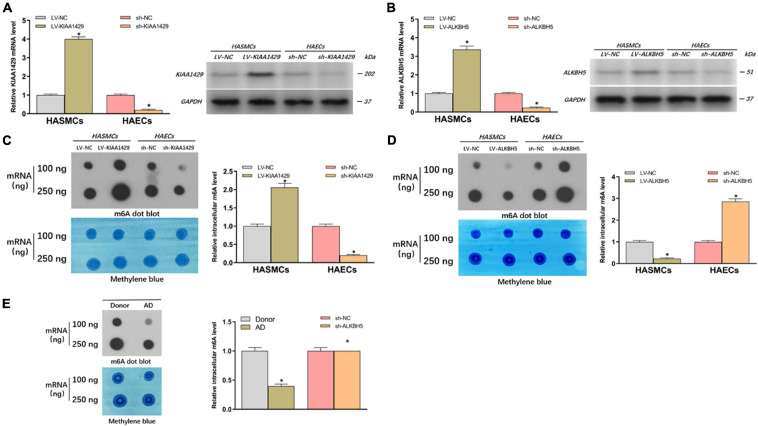
KIAA1429 enhances while ALKBH5 reduces m6A levels in HASMCs and HAECs. **(A,B)** The interference effects on KIAA1429 and ALKBH5 expression in HASMCs and HAECs were detected by qRT-PCR and Western blot. **(C)** Dot blot assays showed that overexpression or silencing of KIAA1429 significantly increased and decreased the level of m6A, respectively. **(D)** ALKBH5 could negatively regulate intracellular m6A levels. **(E)** The m6A level was lower in the aortic tissues from AD patients compared with donors. Data are presented as the mean ± SEM (*N* ≥ 3 per group); *p < 0.05.

To further clarify whether m6A modification is indeed related to AD progression, we investigated the effects of abnormally expressed KIAA1429 and ALKBH5 on m6A levels in HASMCs and HAECs. Firstly, cells were stably transfected with interfering lentiviral vectors and the corresponding control vectors. The interference effects were subsequently measured with qRT-PCR and Western blot (Figures 2A,B). Then, we measured the changes in intracellular levels by performing dot blot assays and observed that overexpression or silencing of KIAA1429 significantly increased and decreased the level of m6A, respectively (Figure 2C). On the contrary, we found that ALKBH5 could negatively regulate intracellular m6A levels (Figure 2D). Moreover, we explored whether there were differences between the m6A levels of AD patients and donors. The results showed that the m6a level was lower in the aortic tissues from AD patients compared with donors (Figure 2E). Collectively, our findings revealed that KIAA1429 and ALKBH5 could influence the development of AD by regulating m6A levels in HASMCs and HAECs.

### KIAA1429 Can Promote HASMC Proliferation, Inhibit HAEC Apoptosis, and Inhibit AD Progression in AngII-Infused Mice

**FIGURE 3:**
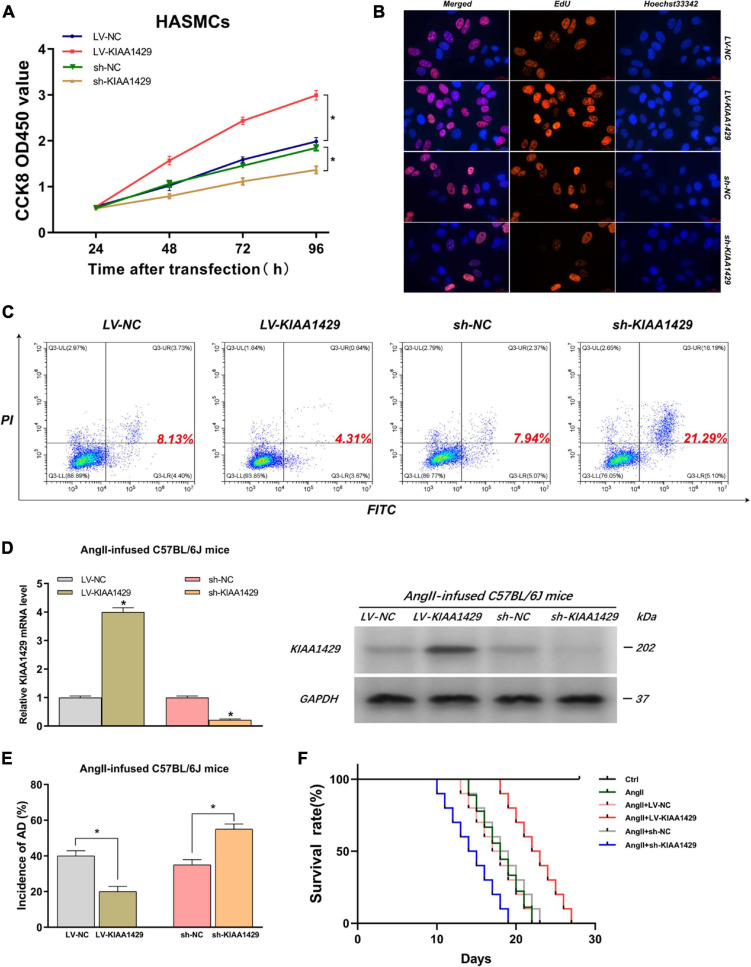
KIAA1429 can promote HASMC proliferation, inhibit HAEC apoptosis, and inhibit AD progression in AngII-infused mice. **(A,B)** KIAA1429 overexpression led to markedly enhanced HASMC proliferation while its knockdown resulted in significantly decreased HASMC proliferation. **(C)** Flow cytometry analysis revealed that overexpression of KIAA1429 notably promoted HAEC apoptosis while knockdown of it significantly inhibited HAEC apoptosis. **(D)** The interference effects on KIAA1429 expression in AngII-infused C57BL/6J mice were detected by qRT-PCR and Western blot. **(E)** KIAA1429 overexpression reduced incidence while its knockdown increased the incidence of AD in AngII-infused mice. **(F)** Mice in the LV-KIAA1429 group and sh-KIAA1429 group had longer and shorter survival times compared to the control group, respectively. Data are presented as the mean ± SEM (*N* ≥ 3 per group); *p < 0.05.

Because the aberrant expression of KIAA1429 in AD tissues had been identified by us, we then assessed the influences of KIAA1429 on the development of AD, including the effects on HASMC proliferation, HAEC apoptosis, and the incidence of AD in AngII-infused mice. Firstly, CCK8 and EdU assays were performed to investigate whether KIAA1429 is involved in HASMC proliferation. Both results indicated that KIAA1429 overexpression led to markedly enhanced HASMC proliferation while its knockdown resulted in significantly decreased HASMC proliferation (Figures 3A,B and Supplementary Figure 7A). Furthermore, the flow cytometry analysis revealed that overexpression of KIAA1429 notably promoted HAEC apoptosis while knockdown of it significantly inhibited HAEC apoptosis (Figure 3C).

To examine whether KIAA1429 affects the development of AD *in vivo*, we interfered with KIAA1429 expression in AngII-infused C57BL/6J mice by injecting AAV9 vectors harboring overexpression plasmids (termed as LV-KIAA1429), shRNA (termed as sh-KIAA1429), or corresponding NC through the tail vein and confirmed interference effect by measuring the mRNA and protein levels of KIAA1429 in arterial tissues of mice (Figure 3D). At 28 days after AngII infusion, the incidence of AD and the survival time of mice in different groups were counted and analyzed. The results indicated that KIAA1429 overexpression reduced the incidence while its knockdown increased the incidence of AD in AngII-infused mice (Figure 3E). Moreover, survival analysis results showed that mice in the LV-KIAA1429 group (*n* = 25) and sh-KIAA1429 group (*n* = 25) had longer and shorter survival times compared to the control group (*n* = 25), respectively (Figure 3F). Taken together, these data revealed that KIAA1429 can promote the development of AD *in vivo* and *in vitro*.

### ALKBH5 Can Suppress HASMC Proliferation, Promote HAEC Apoptosis, and Facilitate AD Progression in AngII-Infused Mice

**FIGURE 4:**
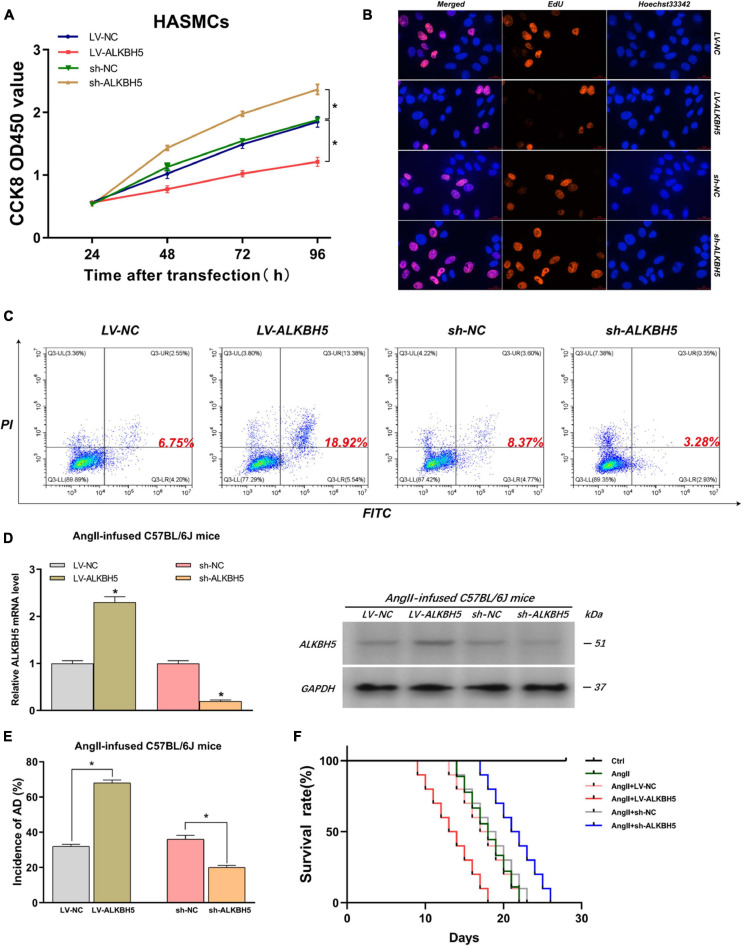
ALKBH5 can suppress HASMC proliferation, promote HAEC apoptosis, and facilitate AD progression in AngII-infused mice. **(A,B)** Both CCK8 and EdU assays found that overexpression of ALKBH5 significantly inhibited HASMC proliferation while knockdown of ALKBH5 led to increased HASMC proliferation. **(C)** Flow cytometry analysis showed that ALKBH5 overexpression notably promoted HAEC apoptosis while its knockdown significantly suppressed HAEC apoptosis. **(D)** The interference effects on ALKBH5 expression in AngII-infused C57BL/6J mice were examined. **(E)** KIAA1429 overexpression reduced the incidence while its knockdown increased the incidence of AD in AngII-infused mice. **(F)** Mice in the sh-ALKBH5 group and LV-ALKBH5 group had longer and shorter survival times compared to the control group, respectively. Data are presented as the mean ± SEM (*N* ≥ 3 per group); *p < 0.05.

By the same token, we explored the impacts of ALKBH5 on AD progression. Firstly, we performed CCK8 and EdU assays and found that overexpression of ALKBH5 significantly inhibited HASMC proliferation while knockdown of ALKBH5 led to increased HASMC proliferation (Figures 4A,B and Supplementary Figure 7B). Subsequently, the flow cytometry analysis showed that ALKBH5 overexpression notably promoted HAEC apoptosis while its knockdown significantly suppressed HAEC apoptosis (Figure 4C). Furthermore, in animal experiments, we first confirmed the effect of interference on ALKBH5 expression and then found that ALKBH5 overexpression could increase the incidence of AD in mice and shorten the survival time of mice (Figures 4D–F). Collectively, these data suggested that ALKBH5 could promote the development of AD *in vivo* and *in vitro*.

### KIAA1429 and ALKBH5 Oppositely Regulate the Processing of miR-143-3p *via* m6A Modification

**FIGURE 5:**
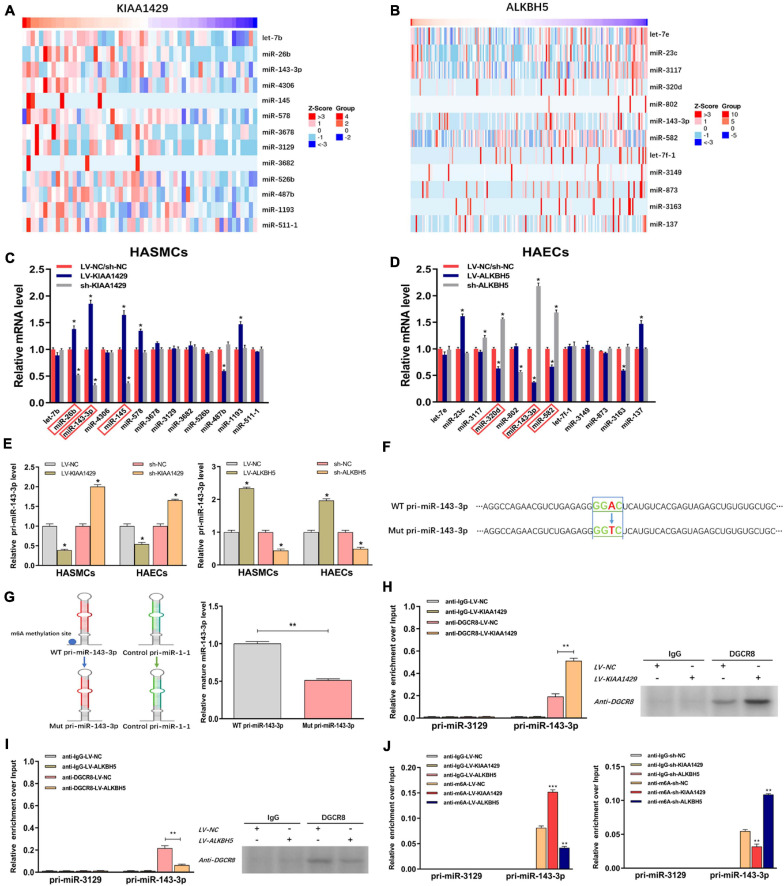
KIAA1429 and ALKBH5 oppositely regulate the processing of miR-143-3p *via* m6A modification. **(A,B)** Through searching in the LinkedOmics database, we found 13 miRNAs positively associated in expression with KIAA1429 and 12 miRNAs negatively associated with ALKBH5. **(C)** The levels of miR-26b, miR-143-3p, and miR-145 increased when KIAA1429 was overexpressed and decreased when KIAA1429 was silenced. **(D)** The levels of miR-320d, miR-143-3p, and miR-582 were negatively regulated by ALKBH5. **(E)** The unprocessed pri-miRNA-143-3p was accumulated in KIAA1429-knockdown or ALKBH5-overexpression cells and significantly decreased in KIAA1429-overexpression or ALKBH5-silence cells. **(F)** In one reporter, the adenosine [A] of the RRACH m6A sequence motif located in the pri-miR-143-3p region outside the pre-miR-143-3p sequence was mutated to thymidine [T]. **(G)** The mutation of the RRACH m6A motif in pri-miR-143-3p significantly reduced its processing to the mature form. **(H,I)** The levels of pri-miR-143-3p bound to DGCR8 were significantly enhanced or reduced by overexpression of KIAA1429 or ALKBH5, respectively. **(J)** MeRIP assay showed that overexpressing KIAA1429 or silencing ALKBH5 notably increased the quantity of pri-miR-143-3p modified by m6A. Data are presented as the mean ± SEM (*N* ≥ 3 per group); *p < 0.05, **p < 0.01, and ***p < 0.001.

Two recent studies have shown that m6A modification can influence tumor progression by affecting the binding of DGCR8 to pri-miRNAs (Lai et al., 2019; Song et al., 2019). Therefore, we herein assessed whether KIAA1429 and ALKBH5 also regulate the combination of DGCR8 and pri-miRNAs in HASMCs and HAECs. Given that enhanced m6A levels can cause the upregulation of miRNAs through facilitating the corresponding pri-miRNA processing (Lai et al., 2019; Song et al., 2019), and that KIAA1429 can improve the m6A levels in HASMCs and HAECs (Figure 2C), we inferred that the levels of KIAA1429/m6A-dependent miRNAs should be positively correlated with KIAA1429. Conversely, the levels of ALKBH5/m6A-dependent miRNAs should be negatively correlated with ALKBH5. Through searching the LinkedOmics database, we found 13 miRNAs positively associated in expression with KIAA1429 and 12 miRNAs negatively associated with ALKBH5 (Figures 5A,B). To confirm whether these candidate miRNAs are regulated by KIAA1429 or ALKBH5 in HASMCs and HAECs, we first measured the changes in their expression levels and found that only miR-26b, miR-143-3p, and miR-145 increased when KIAA1429 was overexpressed and decreased when KIAA1429 was silenced (Figure 5C and Supplementary Figure 2A). Similarly, we observed that only the levels of miR-320d, miR-143-3p, and miR-582 were negatively regulated by ALKBH5 (Figure 5D and Supplementary Figure 2B). We then selected miR-143-3p, the intersection of the above results, for further study.

Subsequently, we determined the level of variation of immature miRNA, pri-miR-143-3p, when the level of KIAA1429 or ALKBH5 changed and observed that unprocessed pri-miRNA-143-3p was accumulated in KIAA1429-knockdown or ALKBH5-overexpression cells and significantly decreased in KIAA1429-overexpression or ALKBH5-silence cells (Figure 5E). To explore the specific mechanisms of this phenomenon, we first searched the m6AVar database for the predicted m6A sites in pri-miR-143-3p, and two RRACH m6A sequence motifs, (GGAC) base sequence, in the pri-miR-143-3p region (only one sequence motif presents outside the pre-miRNA region) were found (Supplementary Figure 3). Then, we recomposed a previously described ectopic reporter construct (Sun et al., 2020). In one reporter, a WT version of pri-miR-143-3p was introduced. In the other reporter, the adenosine [A] of the RRACH m6A sequence motif located in the pri-miR-143-3p region outside the pre-miR-143-3p sequence was mutated to thymidine [T] (Figure 5F). Our findings demonstrated that mutation of the RRACH m6A motif in pri-miR-143-3p significantly reduced its processing to the mature form (Figure 5G).

We next performed the RIP assay to determine the influences of m6A modification on the binding of DGCR8 to pri-miR-143-3p. The results revealed that the levels of pri-miR-143-3p bound to DGCR8 were significantly enhanced or reduced by overexpression of KIAA1429 or ALKBH5, respectively (Figures 5H,I). Moreover, we performed m6A RNA immunoprecipitation (MeRIP) assay and found that overexpressing KIAA1429 or silencing ALKBH5 notably increased the quantity of pri-miR-143-3p modified by m6A (Figure 5J). Taken together, our findings revealed that KIAA1429 could facilitate pri-miR-143-3p processing by enhancing the binding of DGCR8 to it, and ALKBH5 could hinder the processing in the same way.

### MiR-143-3p Is a Downstream Target of KIAA1429 and ALKBH5

**FIGURE 6:**
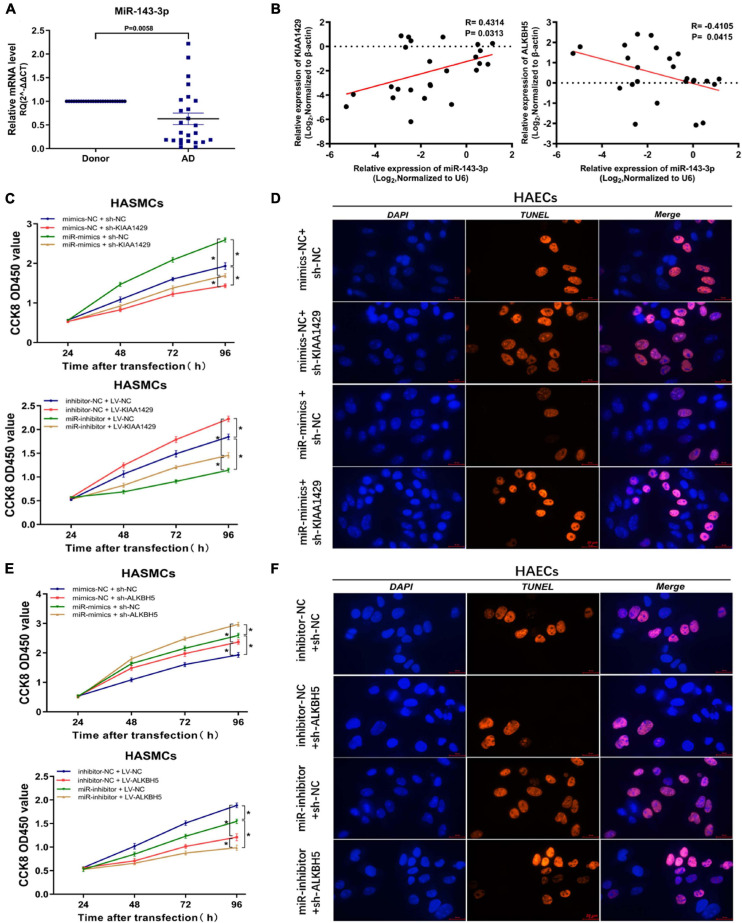
MiR-143-3p is a downstream target of KIAA1429 and ALKBH5. **(A)** MiR-143-3p is significantly downregulated in aortic tissues from AD patients. **(B)** A positive correlation between miR-143-3p and KIAA1429 (*R* = 0.4314, *p* = 0.0313) and a negative correlation between miR-143-3p and ALKBH5 (*R* = –0.4105, *p* = 0.0415) were identified by Pearson’s correlation coefficient analysis. **(C)** MiR-143-3p upregulation could partly restore the cell proliferation suppressed by KIAA1429 knockdown and downregulation of miR-143-3p could partly decrease cell multiplication promoted by KIAA1429 overexpression. **(D)** TUNEL apoptosis assay showed that enrichment of miR-143-3p could partly attenuate HAEC apoptosis induced by the silence of KIAA1429. **(E,F)** Upregulation of miR-143-3p could enhance the pro-proliferative effect of ALKBH5 knockdown on HASMCs and downregulation of miR-143-3p could weaken the anti-apoptotic effect of ALKBH5 knockdown on HAECs. Data are presented as the mean ± SEM (*N* ≥ 3 per group); *p < 0.05.

To investigate the underlying mechanism of miR-143-3p function in AD development as a downstream target of KIAA1429 and ALKBH5, we first examined its expression level in 25 pairs of aorta samples derived from donors and AD patients and found that miR-143-3p was significantly downregulated in tissues from AD patients (Figure 6A). Meanwhile, a positive correlation between miR-143-3p and KIAA1429 (*R* = 0.4314, *p* = 0.0313) and a negative correlation between miR-143-3p and ALKBH5 (*R* = −0.4105, *p* = 0.0415) were identified by Pearson’s correlation coefficient analysis (Figure 6B).

Subsequently, we found that miR-143-3p upregulation could partly restore the cell proliferation suppressed by KIAA1429 knockdown. Accordingly, downregulation of miR-143-3p could partly decrease cell multiplication promoted by KIAA1429 overexpression (Figure 6C). Moreover, TUNEL apoptosis assay showed that enrichment of miR-143-3p could partly attenuate HAEC apoptosis induced by the silence of KIAA1429 (Figure 6D and Supplementary Figure 7C). We then applied the same methods to confirm that increased miR-143-3p could enhance the pro-proliferative effect of ALKBH5 knockdown on HASMCs and decreased miR-143-3p could weaken the anti-apoptotic effect of ALKBH5 knockdown on HAECs (Figures 6E,F and Supplementary Figure 7D).

### MiR-143-3p Directly Targets DDX6 in HASMCs and HAECs

**FIGURE 7:**
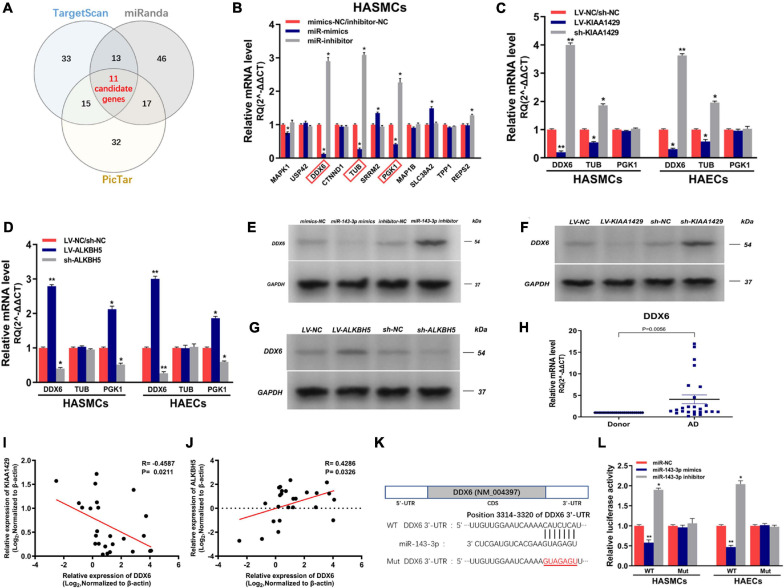
MiR-143-3p directly targets DDX6 in HASMCs and HAECs. **(A)** We searched for putative targets using three databases and obtained 11 candidate genes in their intersection. **(B)** The levels of DDX6, TUB, and PGK1 both changed after the upregulation and downregulation of miR-143-3p. **(C)** The expressions of DDX6 and TUB were reduced or elevated after the overexpression or silence of KIAA1429, respectively. **(D)** Only the levels of DDX6 and PGK1 changed with the upregulation or downregulation of ALKBH5. **(E–G)** Western blot assays confirmed the regulatory effects of miR-143-3p, KIAA1429, and ALKBH5 on the expression levels of DDX6. **(H)** The high expression of DDX6 in aorta samples from AD patients was confirmed by qRT-PCR. **(I,J)** A negative correlation between DDX6 and KIAA1429 (*R* = –0.4587, *p* = 0.0211) and a positive correlation between it and ALKBH5 (*R* = 0.4286, *p* = 0.0326) were identified by Pearson’s correlation coefficient analysis. **(K)** The 3′-UTR of DDX6 was analyzed and one conserved binding domain for miR-143-3p was identified. **(L)** The variations of miR-143-3p level in the 3′-UTR-Wt group significantly decreased or enhanced the luciferase activity, respectively, while the luciferase activity remained unchanged in the 3′-UTR-Mut group. Data are presented as the mean ± SEM (*N* ≥ 3 per group); *p < 0.05, **p < 0.01.

To identify the target genes of miR-143-3p in HASMCs and HAECs, we first searched for putative targets using three databases (TargetScan, miRanda, and PicTar) and obtained 11 candidate genes in their intersection (Figure 7A). Subsequently, we validated the prediction results by qRT-PCR and found that only the levels of DDX6, TUB, and PGK1 both changed after the upregulation and downregulation of miR-143-3p (Figure 7B and Supplementary Figure 4). We next explored whether their levels were affected by KIAA1429 and ALKBH5. The results showed that only the expressions of DDX6 and TUB were reduced or elevated after the overexpression or silence of KIAA1429, respectively (Figure 7C). Meanwhile, only the levels of DDX6 and PGK1 changed with the upregulation or downregulation of ALKBH5 (Figure 7D). Therefore, we choose DDX6, the intersection of these results, as our research object in this study. Consistent with the above qRT-PCR results, subsequent Western blot assays confirmed the regulatory effects of miR-143-3p, KIAA1429, and ALKBH5 on the expression levels of DDX6 (Figures 7E–G). Moreover, we also found the high expression of DDX6 in aorta samples from AD patients, which was negatively correlated with the expression levels of KIAA1429 and positively correlated with ALKBH5 (Figures 7H–J).

Lastly, dual-luciferase reporter assay was performed to substantiate the direct interaction between miR-143-3p and DDX6. The 3′-UTR of DDX6 was analyzed and one conserved binding domain for miR-143-3p was identified (Figure 7K). Then, the wild-type 3′-UTR (Wt) and the mutant 3′-UTR sequence (Mut) of DDX6 were cloned to construct reporter plasmids and mutant vectors, respectively. We found that upregulation or downregulation of miR-143-3p in the 3′-UTR-Wt group significantly decreased or enhanced the luciferase activity, respectively, while the luciferase activity remained unchanged in the 3′-UTR-Mut group, which revealed the targeted binding relationship between miR-143-3p and DDX6 (Figure 7L). Collectively, our findings demonstrated that DDX6 is a direct target gene of miR-143-3p and regulated by both KIAA1429 and ALKBH5.

### KIAA1429 and ALKBH5 Affect AD Progression *via* the miR-143-3p/DDX6 Pathway

**FIGURE 8:**
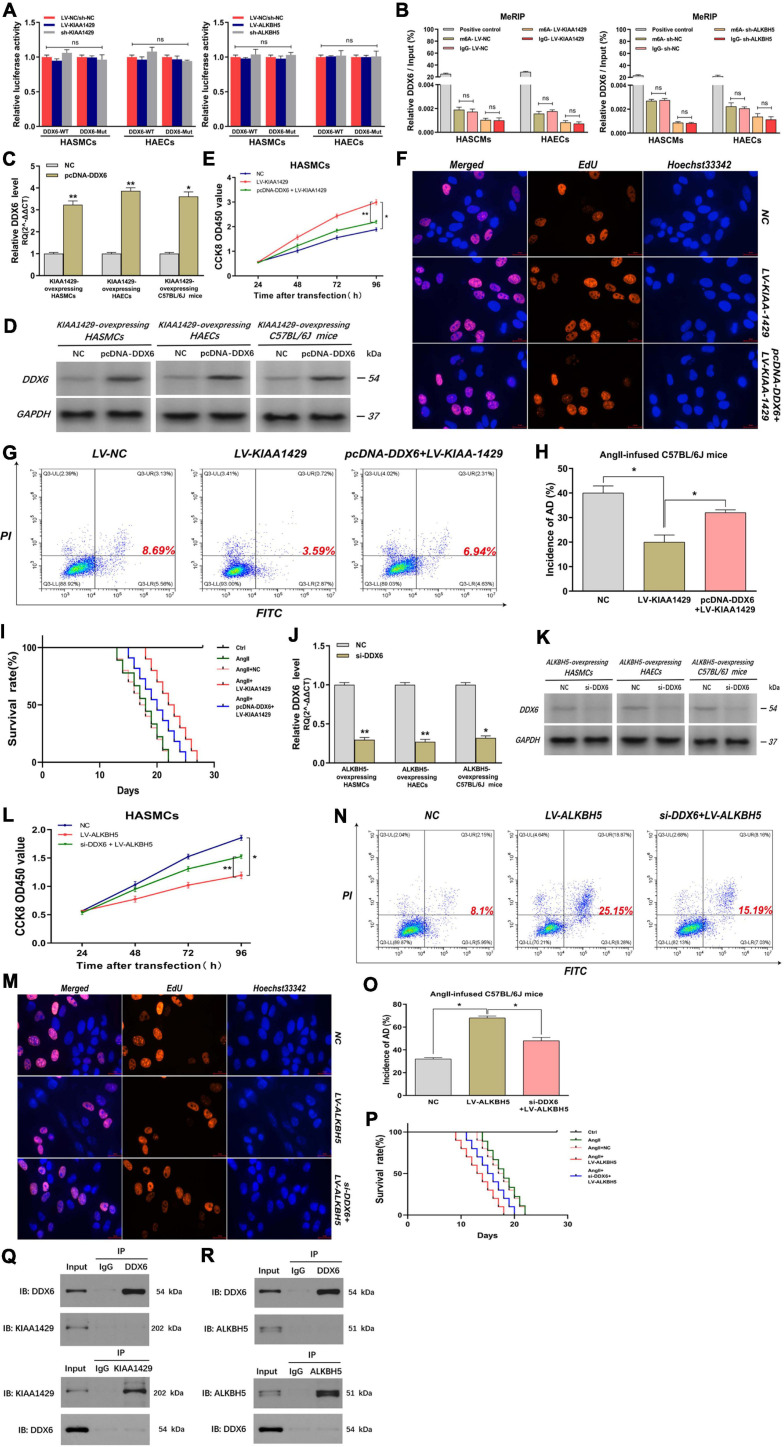
KIAA1429 and ALKBH5 affect AD progression *via* the miR-143-3p/DDX6 pathway. **(A,B)** After the binding site of the 3′-UTR region of DDX6 was mutated, both the luciferase activity and the amount of DDX6 precipitated by m6A and IgG did not change significantly when KIAA1429 or ALKBH5 was disturbed. **(C,D)** The effect of elevating DDX6 level in KIAA1429-overexpressing HASMCs, HAECs, and AngII-infused mice was detected. **(E–I)** The enrichment of DDX6 could partly counteract the effect of the pro-proliferative effect on HASMCs, the anti-apoptotic effect on HAECs, and the inhibitory effect on AD progression in mice induced by KIAA1429 overexpression. **(J,K)** The effect of reducing DDX6 level in ALKBH5-overexpressing HASMCs, HAECs, and AngII-infused mice was examined. **(L–P)** The reduction of DDX6 could partly counteract the effect of the anti-proliferative effect on HASMCs, the pro-apoptotic effect on HAECs, and the promotional effect on AD progression in mice induced by KIAA1429 overexpression. **(Q,R)** Neither KIAA1429 nor ALKBH5 could directly bind to DDX6. Data are presented as the mean ± SEM (*N* ≥ 3 per group); *p < 0.05, **p < 0.01, and ns *p* > 0.05.

Since we had demonstrated that KIAA1429 or ALKBH5 could regulate DDX6 indirectly by modulating miR-143-3p, we intended to further explore whether they could directly regulate DDX6. We performed a luciferase reporter assay and MeRIP assay after the binding site of the 3′-UTR region of DDX6 was mutated. Results indicated that there was no significant change in luciferase activity between groups Wt and Mut, and the amount of DDX6 precipitated by m6A and IgG also did not change significantly when KIAA1429 or ALKBH5 was disturbed (Figures 8A,B and Supplementary Figure 5). These data suggested that neither KIAA1429 nor ALKBH5 may directly regulate DDX6 mRNA in HASMCs and HAECs.

Given that we had clarified the expression relationship between DDX6 and KIAA1429 or ALKBH5, we further explored the functional relationship between them. Through elevating the level of DDX6 in KIAA1429-overexpressing HASMCs, HAECs, and AngII-infused mice (Figures 8C,D), we found that the enrichment of DDX6 could partly counteract the effect of pro-proliferative effect on HASMCs, the anti-apoptotic effect on HAECs, and the inhibitory effect on AD progression in mice induced by KIAA1429 overexpression (Figures 8E–I and Supplementary Figure 7E). Similarly, by reducing the level of DDX6 (Figures 8J,K), we found that DDX6 knockdown also partially neutralized the role of ALKBH5 overexpression in the progression of AD *in vitro* and *in vivo* (Figures 8L–P and Supplementary Figure 7F). Furthermore, we also explored whether KIAA1429 and ALKBH5 also exert their functions through direct binding to DDX6 by performing the co-immunoprecipitation (Co-IP) assay. The results showed that neither KIAA1429 nor ALKBH5 could directly bind to DDX6 (Figures 8Q,R). Taken together, our findings indicated that the miR-143-3p/DDX6 pathway is the downstream effector of KIAA1429 and ALKBH5 affecting AD progression.

## Discussion

The biological functions and mechanisms of various chemical modifications of DNA and protein have been extensively investigated and elucidated (Gawinecka et al., 2017; Tchana-Sato et al., 2018; Song et al., 2019). Nonetheless, the role of RNA modifications remains mostly undiscovered, among which m6A methylation is the most prevalent modification on eukaryotic RNAs. With the advancement of m6A sequence technology, m6A modification has gained increasing attention in recent years (Lai et al., 2019; Sun et al., 2020). Numerous studies revealed that dynamic m6A modifications closely involve multiple physiological and biochemical processes, including maturation of pri-miRNA, transcription splicing, RNA stability, fate and functions of lncRNAs, and RNA–protein interactions (Berger, 2002; Cantara et al., 2011). Based on these findings, it is reasonable to speculate that m6A modification may affect the onset and progression of AD. To confirm the validity of our conjecture and to elucidate its underlying molecular mechanism of action, we conducted the present study.

In our study, we initially measured the expression levels of multiple key genes related to m6A, including “writers,” “readers,” and “erasers,” in aortic tissues derived from donors and AD patients to identify the presence or absence of differentially expressed genes. Results showed the significant low expression of KIAA1429, m6A “writer,” and significant high expression of ALKBH5, m6A “eraser,” in aortas from AD patients. Moreover, we also demonstrated that KIAA1429 enhanced while ALKBH5 reduced the m6A levels in HASMCs and HAECs by using m6A dot blot assay. These findings implied that m6A modification mediated by methyltransferase and demethylase is indeed involved in AD progression. Subsequently, results of a series of *in vitro* and *in vivo* functional experiments uncovered an opposite role of KIAA1429 and ALKBH5 in affecting the progression of AD. KIAA1429 can promote HASMC proliferation, suppress HAEC apoptosis, and facilitate AD progression in AngII-infused mice, while ALKBH5 plays the exact opposite role.

Given that we had revealed the role of KIAA1429 and ALKBH5 in AD development, we next intended to elucidate the potential mechanism by which they function. The findings of two recent studies that METTL14 suppresses the metastasis of hepatocellular carcinoma by modulating pri-miRNA processing *via* interacting with DGCR8 (Song et al., 2019), and that METTL3 could positively modulate the pri-miRNA process through interacting with the microprocessor protein DGCR8 (Lai et al., 2019), suggest that m6A modification can influence disease progression by affecting the binding of DGCR8 to pri-miRNAs. Therefore, we herein assessed whether KIAA1429 and ALKBH5 influence AD progression also through the same mechanism. We first screened out several candidate miRNAs whose expression was regulated by KIAA1429 or ALKBH5 using bioinformatics analysis and expression level detection, from which miR-143-3p was selected as the target in this study because its expression was both positively regulated by KIAA1429 and negatively regulated by ALKBH5. Subsequently, we demonstrated that KIAA1429 could enhance the binding of DGCR8 to pri-miR-143-3p, resulting in the overall increase of mature miR-143-3p and the consequent reduction of unprocessed primary miR-143-3p. On the contrary, ALKBH5 attenuated the binding of DGCR8 to pri-miR-143-3p and thus inhibited pri-miR-143-3p processing, leading to the overall decline of mature miR-143-3p. Furthermore, to clarify the functional mechanism of miR-143-3p more clearly, we predicted and verified its target gene and finally identified DDX6 as a direct target gene of miR-143-3p.

To further strengthen the persuasiveness of signaling pathways revealed in this study, we then directly explored the relationship between DDX6 and KIAA1429 or ALKBH5 at the expression and functional levels. In terms of expression level, KIAA1429 and ALKBH5 could down- or upregulate DDX6, respectively, and it was worth noting here that they indirectly regulated DDX6 levels through modulating pri-mir-143-3p processing, rather than through direct binding to DDX6. In terms of the influence on AD progression *in vitro* and *in vivo*, DDX6 was found to be negatively positively correlated with KIAA1429 and positively correlated with ALKBH5. These data demonstrated that KIAA1429 and ALKBH5 could affect AD progression *via* regulating the miR-143-3p/DDX6 pathway.

In addition, we explored whether two common epigenetic mechanisms other than m6A modification are also associated with the abnormal downregulation of miR-143-3p in aorta tissues of AD patients. Firstly, we treated cells with multiple DNA methyltransferase inhibitors and determined their effects on miR-143-3p expression, whereas no significant difference was observed, suggesting that DNA methylation might not be related to miR-143-3p expression in HASMCs and HAECs (Supplementary Figure 6A). Subsequently, we investigated the role of histone acetylation in miR-143-3p expression *via* treating cells with various histone deacetylase (HDAC) inhibitors. The results revealed that these HDAC inhibitors had no significant influence on miR-143-3p level (Supplementary Figure 6B). This conclusion was further confirmed by results showing that the overexpression of HDAC also had no impact on miR-143-3p expression (Supplementary Figure 6C). These findings suggested that neither DNA methylation nor histone acetylation is related to the decline of miR-143-3p, which further highlighted the importance of m6A modification in influencing the onset and progression of AD.

Although we tried our best to design our study as rigorously and completely as possible, there are still limitations. One limitation is that the clinical tissue specimen size needs to be further expanded for reducing the likelihood of bias, such as gender bias and age bias. A major obstacle to overcoming this deficiency is that normal aorta samples are derived from donations in China and the number of heart donors declared brain dead is in short supply. Another shortcoming is that we only investigated the function and mechanism of m6A modification in two cell types with the highest relevance to AD development in this study, HASMCs and HAECs. Its role and potential function mechanism in other cell types in the arterial vessel wall need to be further explored in future studies.

In summary, our findings have revealed a novel connection between m6A modification and AD progression. Concretely, we found that KIAA1429 is downregulated while ALKBH5 is upregulated in aortic tissues from AD patients. We demonstrated that KIAA1429/ALKBH5-mediated m6A modifications can regulate the processing of pri-miR-143-3p through interacting with the microprocessor protein DGCR8. Furthermore, KIAA1429 and ALKBH5 can oppositely regulate HASMC proliferation, HAEC apoptosis, and AD progression in AngII-infused mice *via* the miR-143-3p/DDX6 pathway. The findings presented here reveal the role of m6A modification in AD progression for the first time and may provide a novel molecular basis for subsequent researchers to search for novel therapeutic approaches to improve the health of patients struggling with AD.

## Data Availability Statement

The original contributions presented in the study are included in the article/Supplementary Material, further inquiries can be directed to the corresponding author/s.

## Ethics Statement

The studies involving human participants were reviewed and approved by Clinical Research Ethics Committees of Renmin Hospital of Wuhan University. The patients/participants provided their written informed consent to participate in this study. The animal study was reviewed and approved by The Ethical Committee of the Renmin Hospital of Wuhan University.

## Author Contributions

PW designed the project plan, completed the main experiment, and drafted the manuscript. ZW contributed to the resources and supervised the implementation of the whole project. MZ organized the experimental data and revised the manuscript. QW and FS established the AngII infusion animal model. SY completed the bioinformatics analysis. All authors have read and approved the final version of the manuscript.

## Conflict of Interest

The authors declare that the research was conducted in the absence of any commercial or financial relationships that could be construed as a potential conflict of interest.

## Publisher’s Note

All claims expressed in this article are solely those of the authors and do not necessarily represent those of their affiliated organizations, or those of the publisher, the editors and the reviewers. Any product that may be evaluated in this article, or claim that may be made by its manufacturer, is not guaranteed or endorsed by the publisher.
