# Supplementary material for: KIAA1429 and ALKBH5 Oppositely Influence Aortic Dissection Progression via Regulating the Maturation of Pri-miR-143-3p in an m6A-Dependent Manner
Source: Front Cell Dev Biol. 2021 Aug 19;9:668377. doi: 10.3389/fcell.2021.668377 (PMC8416753; doi:10.3389/fcell.2021.668377)
Supplement: Supplementary file 1 [file Data_Sheet_1.PDF]

Supplementary Figures and Tables

Supplementary Figure. 1

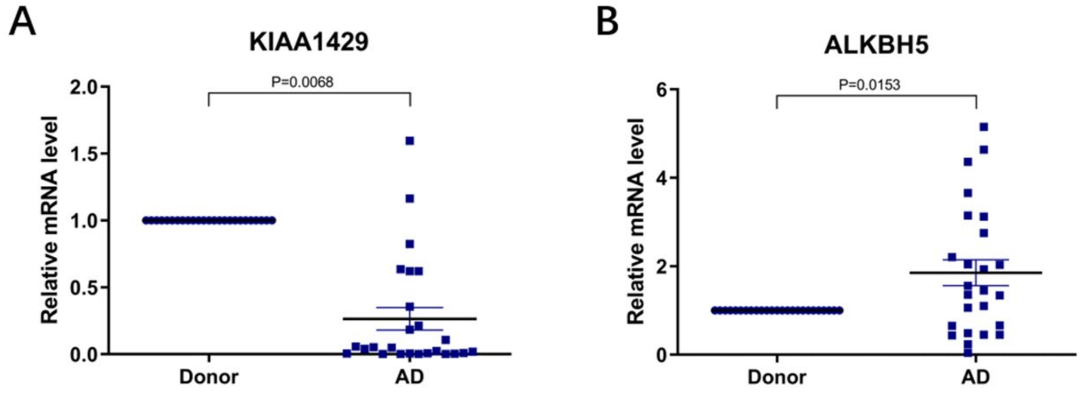

The changes in the expression levels of KIAA1429 and ALKBH5 in clinical samples. (A-B) The low expression of KIAA1429 or high-expression of ALKBH5 was confirmed in 25 pairs of aortic tissues from AD patients compared with donors. Data are presented as the mean ± SEM (N⩾3 per group); *P < 0.05.

Supplementary Figure. 2

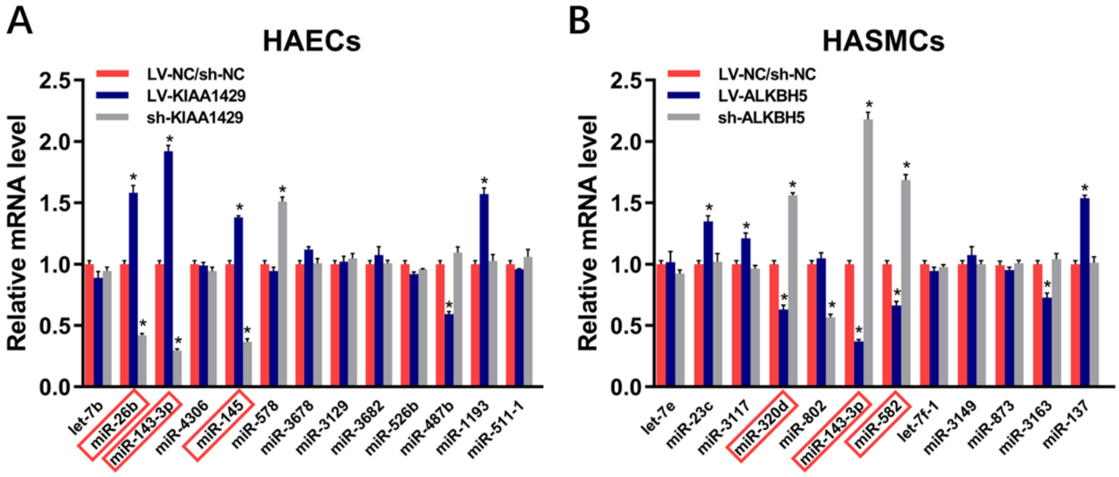

The changes in the expression levels of candidate miRNAs were examined. (A) MiR-26b, miR-143-3p, and miR-145 increased when KIAA1429 was overexpressed and decreased when KIAA1429 was silenced in HAECs. **(B)** MiR-320d, miR-143-3p, and miR-582 were reduced when ALKBH5 was overexpressed and increased when ALKBH5 was silenced in HASMCs. Data are presented as the mean ± SEM (N⩾3 per group); *$P < 0.05$.

Supplementary Figure. 3

pri-miR-143

GGUGCAACCAGAGGAGGGCCAGCAGCAGGCAGCCCGGAGACGUGCUGCAU
CUCUGGUAUGCUGAUGUCAGAGAAGCACAAACAGGCUGGCUCCCGUCUCC
AGGCCAGAACGUCUGAGAGG**GGAC**UCAUGUCACGAGUAGAGCUGUGUG
CUGCAUCUCCGCCCCCCAGGU

pre-miR-143

AGACGACGUGCUGCAAGACAGCGCAGCGCCCUGUCUCCCAGCCUGACAGU
UG**GGAC**UCUGAGAUGAAGCACUGUAGCUCAGGAAGAGAGAAGUUGUUC
UGCAGC

pri-miR-143-3p

GUGACACCCCCAACCACUCCCCAAACAGGCUGGCUCCCGUCUCCAGGCCC
CAAGGAGCCACACCUGGAUCAGACCCCAGGAAAGGCAAGGGCGCUAGCUG
GUGGGAGCCACCCCGCCAUGCUGAUGUCAGAGAAGCAAGAACUCUGGAGA
AGCAGCCUCCUGGUACCAGAGGAGGGCCAGCAGCAGGCAGCCCGG

Two RRACH m6A sequence motifs, [GGAC] base sequence, in pri-miR-143-3p region (only one sequence motif presents outside the pre-miRNA region), were found.

Supplementary Figure. 4

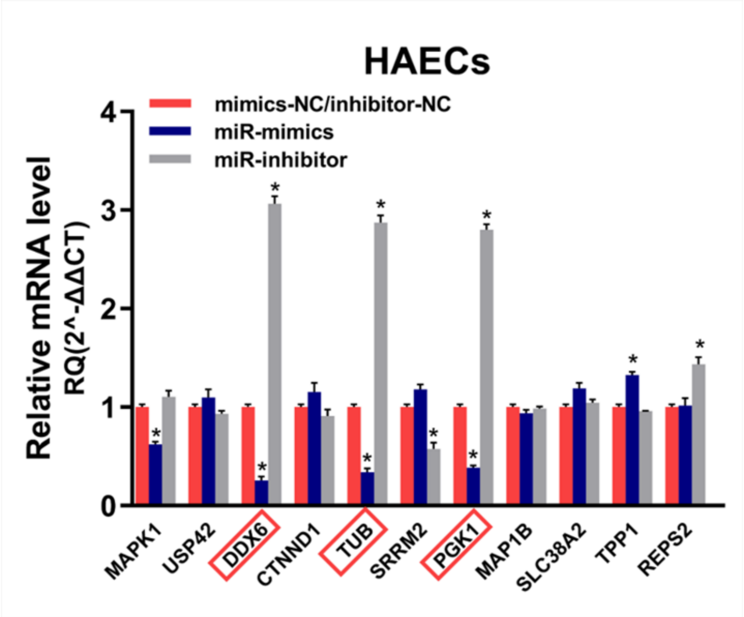

Only the levels of DDX6, TUB, and PGK1 both changed after the upregulation and downregulation of miR-143-3p in HAECs. Data are presented as the mean ± SEM (N⩾3 per group); *P < 0.05.

Supplementary Figure. 5

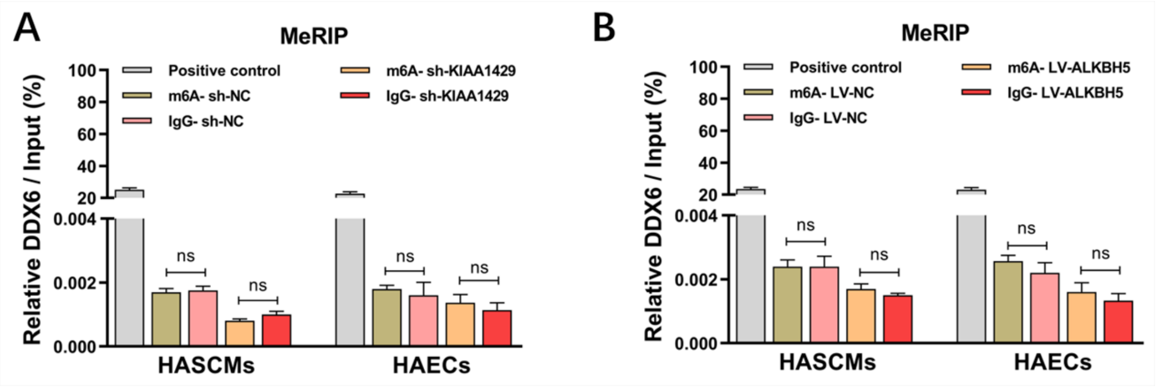

After the binding site of the 3′-UTR region of DDX6 was mutated, the amount of DDX6 precipitated by m6A and IgG did not change significantly when KIAA1429 or ALKBH5 was disturbed in HASMCs and HAECs. Data are presented as the mean ± SEM (N⩾3 per group); ns P > 0.05.

Supplementary Figure. 6

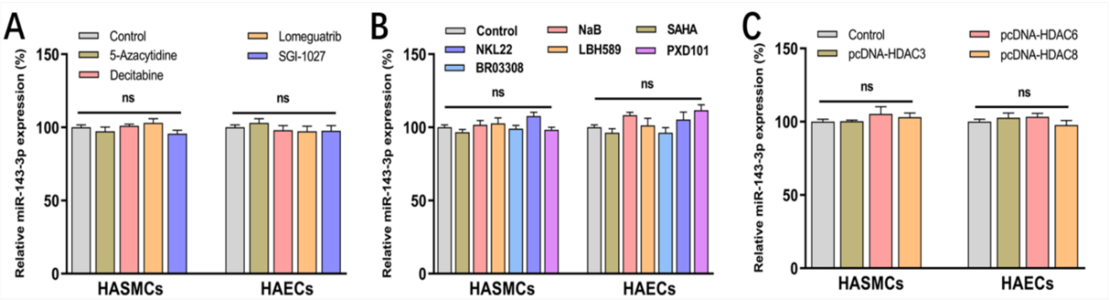

Neither DNA methylation nor histone acetylation is related to the decline of miR-143-3p. (A) By treating cells with multiple DNA methyltransferase inhibitors and examining their effects on miR-143-3p expression, we found no significant difference between groups. **(B)** HDAC inhibitors had no significant influence on the miR-143-3p level. **(C)** The overexpression of HDAC also had no impact on miR-143-3p expression. Data are presented as the mean ± SEM (N⩾3 per group); ns $P > 0.05$.

Supplementary Figure. 7

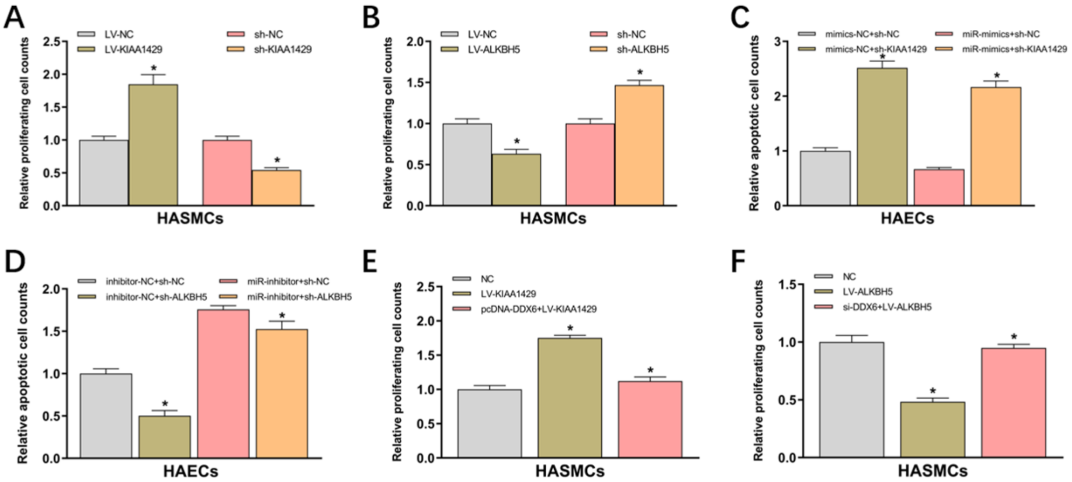

Statistical analysis of the corresponding EdU proliferation and TUNEL apoptosis experiments in the main text. (A) Results of statistical analysis of Fig 3B. **(B)** Results of statistical analysis of Fig 4B. **(C)** Results of statistical analysis of Fig 6D. **(D)** Results of statistical analysis of Fig 6F. **(E)** Results of statistical analysis of Fig 8F. **(F)** Results of statistical analysis of Fig 8M. Data are presented as the mean ± SEM (N⩾3 per group); ns $P > 0.05$.

Supplementary Table. 1

Clinical characteristics of AD patients and donors enrolled in this study

	Donor group (n =25)	AD group (n = 25)	*P*-value
Ages (year)	49.6 ± 7.5	50.8 ± 10.1	0.338
Male (n)	16	18	0.762
Smoking history (n)	10	13	0.570
Drinking history (n)	8	11	1.000
Hypertension (n)	5	17	**0.002
Diabetes mellitus (n)	2	5	0.415
SBP (mmHg)	127.9 ± 12.3	148.2 ± 17.1	*0.018
DBP (mmHg)	79.5 ± 5.4	93.2 ± 7.4	0.118
Fasting blood glucose(mmol/L)	6.01 ± 0.8	8.5 ± 2.8	0.894
TC (mmol/L)	4.07 ± 0.5	4.35 ± 0.8	0.938

SBP, systolic blood pressure; DBP, diastolic blood pressure; TC, total cholesterol. *$P < 0.05$ and **$P < 0.01$.

Supplementary Table. 2

The primer sequences used in the present study

Name		Sequences
METTL3	Sense	5'- CAAGCTGCACTTCAGACGAA-3'
	Anti-sense	5'- GCTTGGCGTGTGGTCTTT-3'
METTL14	Sense	5'- AGAAACTTGCAGGGCTTCCT -3'
	Anti-sense	5'- TATCCCTCTTGGTCTGTGGAG-3'
WTAP	Sense	5'- GGCGAAGTGTCGAATGCT -3'
	Anti-sense	5'- CCAACTGCTGGCGTGTCT -3'
KIAA1429	Sense	5'- GAAGGCTGGAGTTGCTTGAG -3'
	Anti-sense	5'- ACCCTTGTCCAAACATGCAC -3'
MET16	Sense	5'- GAACCAGGTGGCCCTAGATT -3'
	Anti-sense	5'- AACGGAGGAACGGTATTGGT -3'
RBM15B	Sense	5'- GCAGCCCTGTCCATTGTATG -3'
	Anti-sense	5'- TGACAGTAGGAAGCAGCACT -3'
YTHDC1	Sense	5'- AGATCCTGGGATGAGGAGGT -3'
	Anti-sense	5'- AGAGCCAGGAGGATTTGGAC -3'
YTHDF1	Sense	5'- CATTCCTGTGGGTTCTTGGT -3'
	Anti-sense	5'- CATCTGAAGGAACAAACTCCGGA-3'
HNRNP	Sense	5'- CATTCCTGTGGGTTCTTGGT -3'

	Anti-sense	5'- GAAGTACGGCACCAGCATTT -3'
eIF3	Sense	5'- TGCCCTCAAGATGCACATCCG A-3'
	Anti-sense	5'- GGGACAGGAGAAGGGCTTCTC -3'
IGF2BP1	Sense	5'-ATCTGCGGCAAGGCGTTTTCCA -3'
	Anti-sense	5'- GAGCCCTCAGATTTGACCTGTC- 3'
ALKBH5	Sense	5'- CCCGAGGGCTTCGTCAACA -3'
	Anti-sense	5'- CGACACCCGAATAGGCTTGA -3'
FTO	Sense	5'- TGGGTTCATCCTACAACGG -3'
	Anti-sense	5'- CCTCTTCAGGGCCTTCAC -3'
MAPK1	Sense	5'- GAGGAAGATGGATTGGTTGC -3'
	Anti-sense	5'- GCTGGCATAGGAGGATGAAG -3'
USP42	Sense	5'- CTACCAGCCATACGAGATTCC -3'
	Anti-sense	5'- AAGAAGACAAAGAGGCAGAGG- 3'
DDX6	Sense	5'- AACAACCTTAGTTTTGTGAGAGCA -3'
	Anti-sense	5'- TAGTTGGAATCGCCCGACTCTCGT-3'
CTNMD1	Sense	5'- ACTGCCAAGTGAGAGAAGTGG-3'
	Anti-sense	5'- AGGTGCTGTTTCGGTGCTAC- 3'
TUB	Sense	5'- TATGCCTAGTCCAGCCACAG-3'
	Anti-sense	5'- GAGCGAACAAAGCGGTGTAT-3'
SRRM2	Sense	5'- CACCGTGGGATCTGAGAGTA-3'
	Anti-sense	5'- CACGAGGTCTTGCAAGGATG-3'
PGK1	Sense	5'- GGAGACAGGGTTGCCATTG -3'
	Anti-sense	5'- CGCAGAAAGAGTTCCAGCTA -3'
MAP1B	Sense	5'- AGCGAGGTAAAGTTGCGTCT -3'
	Anti-sense	5'- TGGTTTTCTGGGCCATACCG -3'
SLC38A2	Sense	5'-CTCTGAATAGTCCAGTGAGACCT-3'
	Anti-sense	5'-CTGACTCTTAGAGGTAGAATGAGC-3'
TPP1	Sense	5'- CAGCAGATGAATGGAAGTGA -3'
	Anti-sense	5'- TGCGGAAGTATGGCATGTGTC -3'
REPS2	Sense	5'-AAAAGAATGCTTTAGGTGGG-3'
	Anti-sense	5'-AAGGAATACTCCCTGACTTC-3'
GAPDH	Sense	5'- CCACAGTCCATGCCATCAC -3'
	Anti-sense	5'- GCTTCACCACCTTCTTGATG- 3'
U6	Forward	5'- GGAACGATACAGAGAAGATTAGC-3'
	Reverse	5'- CGGAACGCTTCACGAATTTGCG -3'
Let-7b	Forward	5'- GCAACGATACAGTGAAGATTC-3'
	Reverse	5'- TGGAACGCTTCACCAATTTGCG -3'
MiR-26b	Forward	5'- ACCTGCAATTCCTTGCCACTTC -3'
	Reverse	5'- ATGTCGATGATTGCCTGCAC -3'
MiR-143-3p	Forward	5'- TTTCTCAGACGTGCGCAATG -3'
	Reverse	5'- GTTGATGTTGATACAGCTCTC -3'
MiR-4306	Forward	5'- TAACCAGGTGGCCCTAGATA -3'
	Reverse	5'- AACGGAGGAAGCGTATTGGT -3'
MiR-145	Forward	5'- ACAGCCCTGTCCATTGTATG -3'

	Reverse	5'- TGACAGTAGGAAGCAGCACC -3'
MiR-578	Forward	5'- GAGATCCTGGGATGAGGAGGG -3'
	Reverse	5'- CAGAGCCAGGAGGATTTGGAC -3'
MiR-3678	Forward	5'- CAATCCTGTGGGTTCTTGGT -3'
	Reverse	5'- TATGTGAAGGAACAAACTCCGGA-3'
MiR-3129	Forward	5'- ATTCCTGTGGGTTCTTGGCCT -3'
	Reverse	5'- GAAGTACGGCACCAGCATTT -3'
MiR-3682	Forward	5'- CTGCCCTGTCATGCACATAGTA-3'
	Reverse	5'- CGGACAGGAGAACGGCTTCTC -3'
MiR-526b	Forward	5’-ATCTGCGGCAAGGCGTTATCCG -3’
	Reverse	5’- GAGCCCTCTGATTTGACCTGTC- 3’
MiR-487b	Forward	5'- CGAACGATACAGAGAAGATTCG-3'
	Reverse	5'- ATGGAAGCTTTCACGAATTTAC -3'
MiR-1193	Forward	5'- CCTGCAATTCCTTGCCACTTG -3'
	Reverse	5'- GATGTCCATGATTGCCTGCAC -3'
MiR-511-1	Forward	5'- TGAGGGCTGGAGTTGCTTGAC -3'
	Reverse	5'- ATCCTTGTCCAAACATGCAG -3'
Let-7e	Forward	5'- TAACCACGTGGCCCTAGATC -3'
	Reverse	5'- AACGGTGGAACGGTATTGGG -3'
MiR-23c	Forward	5'- GCAGTCCTGTCCATTGTATG -3'
	Reverse	5'- CGACAGTAGGAAGCCGCAC -3'
MiR-3117	Forward	5'- GATCCTGGGATGAGGAGGTG -3'
	Reverse	5'- CAGAGCCAGGAGGATTTGCAC -3'
MiR-320d	Forward	5'- CATTCCTGTGGGTTCTTGGTG -3'
	Reverse	5'- CGATATGTAGGAACAAACTCC-3'
MiR-802	Forward	5'- AATTCCTGTGGGTTCTTGGTT -3'
	Reverse	5'- CAAGTACGGCACCAGCATTTA -3'
MiR-582	Forward	5'- AGCCCTCACGATGCACATCCGT-3'
	Reverse	5'- AGGACAGGAGAAGCGCTTCAG -3'
Let-7f-1	Forward	5’-ACTGCGGCAAGGCGTTTTCCAG -3’
	Reverse	5’- AGCCCTCAGATATGACCTGTCG- 3’
MiR-3149	Forward	5'- CGATTCCTGTGAGTTCTTGGTG -3'
	Reverse	5'- GCATCTGAAGGAACAAACTCC-3'
MiR-873	Forward	5'- GATTCATGTGGGTTCTTGGTA -3'
	Reverse	5'- CGAAGTACGGCACCAGCATTTG -3'
MiR-3163	Forward	5'- ATGCGCTCAAGATGCACATCCT-3'
	Reverse	5'- CGGACAGCAGAAGGGCTTCTCG -3'
MiR-137	Forward	5’-TCTGCGGCACGGCGTTTTCCA -3’
	Reverse	5’- GCCCTCAGATTTGACCTGTCT- 3’

Supplementary Table. 3

Primary antibodies used in this study

Name	Manufacturer	Product code	Dilution rate
Anti-KIAA1429	Invitrogen, USA	# PA5-95717	1:10000
Anti-ALKBH5	Abcam, UK	# ab69325	1:500
Anti-DGCR8	Abcam, UK	# ab191875	1:1000
Anti-IgG	Abcam, UK	# ab172730	1:5000
Anti-DDX6	Cell Signaling Technology, UK	# 5246	1:5000
Anti-GAPDH	Abcam, UK	# ab8245	1:5000

Supplementary Table. 4

The databases involved in the bioinformatics analysis in this study.

Database name	Available addresses
LinkedOmics	http://www.linkedomics.org/login.php
m6AVar	http://m6avar.renlab.org/index.html
TargetScan	http://www.targetscan.org/
miRanda	http://www.microrna.org/microrna/home.do
PicTar	http://www.pictar.org/
